# Health literacy, self-efficacy, self-care behaviors, and glycemic control among older adults with type 2 diabetes mellitus: a cross-sectional study in Thai communities

**DOI:** 10.1186/s12877-023-04010-0

**Published:** 2023-05-16

**Authors:** Parichat Ong-Artborirak, Katekaew Seangpraw, Sorawit Boonyathee, Nisarat Auttama, Prakaipetch Winaiprasert

**Affiliations:** 1 Faculty of Public Health, Chiang Mai University, Chiang Mai, Thailand; 2 School of Public Health, University of Phayao, Phayao, Thailand; 3 School of Medicine, University of Phayao, Phayao, Thailand; 4 Faculty of Nursing, Thammasat University, Pathum Thani, Thailand

**Keywords:** Health literacy, Self-efficacy, Behaviors, FBS, HbA1c, Diabetes, Elderly, Thailand

## Abstract

**Background:**

Properly understanding the health information of people with type 2 diabetes mellitus (T2DM) is the basis for better risk factor management, which also positively affects their quality of life. The aim of this study was to investigate diabetes health literacy (HL), self-efficacy, and self-care behaviors associated with glycemic control among older adults with T2DM in northern Thai communities.

**Methods:**

A cross-sectional study was conducted among 414 older adults over the age of 60 diagnosed with T2DM. The study was conducted in Phayao Province from January to May 2022. Simple random sampling of the patient list for the Java Health Center Information System program was used. Questionnaires were used to collect data on diabetes HL, self-efficacy, and self-care behaviors. Blood samples were tested for estimated glomerular filtration rate (eGFR) and glycemic controls, such as fasting blood sugar (FBS) and glycated hemoglobin (HbA1c).

**Results:**

The mean age of participants was 67.1 years. FBS (mean ± SD = 108.5 ± 29.5 mg/dL) and HbA1c (mean ± SD = 6.6 ± 1.2%) levels were found to be abnormal in 50.5% (≥ 126 mg/dL) and 17.4% (≥ 6.5%) of the subjects, respectively. There was a strong correlation between HL and self-efficacy (r = 0.78), HL and self-care behaviors (r = 0.76), and self-efficacy and self-care behaviors (r = 0.84). The eGFR was significantly correlated with diabetes HL (r = 0.23), self-efficacy (r = 0.14), self-care behaviors (r = 0.16), and HbA1c (r = -0.16) scores. Linear regression after adjusting for sex, age, education, DM duration, smoking, and drinking alcohol showed that FBS level was inversely associated with diabetes HL (Beta = -0.21, R^2^ = 11.0%), self-efficacy (Beta = -0.43, R^2^ = 22.2%), and self-care behavior (Beta = -0.35, R^2^ = 17.8%), whereas HbA1C level was negatively associated with diabetes HL (Beta = -0.52, R^2^ = 23.8%), self-efficacy (Beta = -0.39, R^2^ = 19.1%), and self-care behavior (Beta = -0.42, R^2^ = 20.7%).

**Conclusion:**

Diabetes HL was related to self-efficacy and self-care behaviors in elderly T2DM patients and was shown to influence their health, including glycemic control. These findings suggest that implementing HL programs to build competence in self-efficacy expectations is important for contributing to improvements in diabetes preventive care behaviors and HbA1c control.

## Introduction

Type 2 diabetes mellitus (T2DM) is rapidly becoming one of the world’s most concerning non-communicable diseases in public health, and it has negative economic impacts on individuals and healthcare systems in almost every nation [1]. According to the World Health Organization (WHO), in 2016, approximately 422 million adults worldwide were affected with diabetes, and the WHO estimated that this would increase by about 48% by the year 2045 [2]. The National Health Report of Thailand for Physical Examination in 2008–2009 showed that the prevalence of diabetes was highest among the age group 60–69 years (16.7%) and higher in females than males (19.2% vs. 13.6%) [3]. National Statistical Report of the Ministry of Public Health, Thailand (2020) found that the incidence of diabetes is continuously increasing among Thai people [4]. There are approximately 300,000 new cases per year; and 3.2 million people are registered in the diabetes registry database [4]. The report showed that diabetes alone causes huge losses due to healthcare costs, with the average cost of treatment as high as 47.6 billion baht per year [3, 4]. A literature review showed that people with long-term diabetes and inadequate glycemic management were more likely to develop health complications, increasing their likelihood of severe health conditions and death [5]. The main indicator of blood sugar control is glycated hemoglobin (HbA1c) [6]. Lower HbA1c levels are associated with lower mortality rate and lower health complications among people with diabetes [7, 8]. Several studies have suggested that self-care behaviors and good health knowledge are associated with good blood sugar control [9, 10]. Another study pointed out that one of the main determinants of HbA1c control is good self-care behaviors [10].

Several studies have indicated that health literacy (HL) has a significant impact on self-care compliance and diabetes outcomes [11, 12]. Due to the increasing complexity of health systems, knowledge regarding health, accessibility to health information skills, and self-care behaviors are incredibly important for patients [13]. According to the WHO definition, health literacy is an individual’s cognitive and social skills regarding health, which defines their motivation and ability to access, understand, and use information in a way that promotes and maintains good health [14]. Health literacy facilitates a person with T2DM to access necessary health information and enables self-management of their own health [15]. Reisi (2016) showed that health literacy enables patients to engage in health-related behaviors and perform appropriate self-care behaviors [16]. Health literacy is effective for improving health outcomes among diabetic patients [16, 17]. Previous studies have indicated that HL plays an important role in self-care, medication compliance, and clinical outcomes among diabetes patients [18, 19]. However, research on the diabetes HL associated with glycemic control, as well as other health outcomes among T2DM patients in Thailand, an upper-middle-income country, is limited.

Self-efficacy refers to individuals’ belief in their ability to execute necessary behaviors and practices to produce specific performance attainments [20, 21]. Individuals’ behaviors are often predicted by their beliefs about their ability to control their own motivations, behaviors, and social environment [21]. Self-efficacy determines what individuals do with the knowledge and skills related to the expected outcomes. Self-efficacy is one of the determinants of expected outcomes among people with diabetes [22]. A previous study found that self-efficacy is positively associated with self-management of health, blood sugar, and glycemic control among diabetes patients [23].

Self-care behaviors are individuals’ decisions and actions to cope with health problems and to improve health behaviors [24, 25]. Some studies have suggested that self-care behaviors are a determinant of disease control and related health complications [26]. Factors such as knowledge information, physical and emotional skills, self-efficacy, and health perceptions are associated with self-care behaviors among diabetic patients [26]. Diabetes is a complex chronic metabolic disease that requires ongoing medical care, and patients must be responsible for their own self-care behaviors in term of medication compliance, diet, exercise, and other related behaviors [27–29]. Therefore, promoting daily self-care behaviors among people with diabetes is very important for regulating metabolism and eliminating the health complications of diabetes, leading to improved health [30]. In this study, the concepts of self-care behaviors and self-efficacy were used to assess elderly T2DM patients. The objective of this study was to assess diabetes HL, self-efficacy, and self-care behaviors associated with glycemic control and other health outcomes among older adults with T2DM in community areas of northern Thailand. This can be useful for promoting diabetes health planning in order to control abnormal blood glucose levels, raise health awareness, and promote appropriate self-care behaviors to prevent complications and deaths among diabetes patients, enabling multidisciplinary medical and public health teams to collaborate with the community to implement HbA1c control activities tailored to the context of the target area.

## Methods

### Study design and setting

A cross-sectional study was conducted under the Unit of Excellence Project “Health Promotion and Quality of Life” in Muang District, Phayao Province, Northern Thailand. Data were collected from January to May 2021. Health administrators from Health Promoting Hospitals and healthcare personnel provided assistance and support with conducting the research.

### Population and sampling procedures

A simple random sampling method was used. Based on a lottery of 15 sub-districts in Muang District, two subdistricts Ban Tom and Ban Mai were selected for the study. The terrain is characterized by plateaus and low slopes, and each community has limited access to health services. The participants were recruited using simple random sampling based on a list of patients who had received health services at the primary healthcare unit (Health Promoting Hospitals in both of the selected subdistricts) according the Java Health Center Information System (JHCIS) during the years 2020–2021. The inclusion criteria were: (a) females and males ages 60 years and older residing in the area for at least 1 year who (b) had been diagnosed with T2DM by a medical doctor and enrolled in the JHCIS program and (c) did not suffer from cognitive disorders or blindness according to medical records. Those who were unable to communicate in the local language or who did not voluntarily sign the written consent form prior to participating in the research were excluded from the study.

The sample size was calculated by assuming maximum variability or estimating the abnormal HbA1C proportion at 50%, with 5% absolute precision and a 95% confidence level. The study required a sample size of 427 patients based on the calculation, which was increased by 10%. Finally, complete data from 414 participants were evaluated. Prior to conducting the research, five research assistants were recruited for each subdistrict. Two were public health academics, and three were village health volunteers with the skill needed to communicate in the local language and collect information from the research participants.

### Variables

#### Dependent variables

The primary outcomes of this study were glycemic control, such as FBS and HbA1c levels. Additionally, medical examination results, such as body mass index (BMI), systolic blood pressure (SBP), diastolic blood pressure (DBP), and estimated glomerular filtration rate (eGFR) were used to assess the health of elderly T2DM patients.

#### Independent variables

Diabetes HL, self-efficacy, and self-care behaviors were independent variables of interest in this study. Participants’ general characteristics sex, age, marital status, education, employment status, income level, smoking, alcohol intake, favorite food taste, DM duration, comorbidity, medication adherence, and presence of a caregiver were also collected.

### Data collection

The participants signed a consent form before undergoing a physical examination, providing a blood sample, and completing the questionnaire. They were measured for height, weight, and blood pressure. A nurse took a 3-mL blood sample from each elderly patient. On the same day, blood specimens were properly stored and transported to the Phayao Hospital Medical Laboratory Center for laboratory testing. The research assistants conducted a face-to-face interview that lasted about 20–30 min. The questionnaire was developed based on previous studies and literature reviews that were appropriate to the context of elderly people in northern Thailand. The questionnaire consisted of four parts. Part 1 asked about patients’ general characteristics. Part 2 was related to diabetes HL and was modified from previous research [31, 32]. The questions were divided into six areas: (1) accessibility skills regarding health and health services, (2) cognitive skills regarding the symptoms and prevention of diabetes, (3) communication skills regarding awareness of diabetes and its complications, (4) healthy decision-making skills, (5) self-management skills, and (6) social media skills for finding diabetes information. Each domain contained six questions, for a total of 36 questions in Part 2. Participants chose from three possible answers: yes, not sure, and no. A correct answer was equal to 1 point, and an answer of not sure or no was equal to 0 points. The total score was in the range of 0 to 36 points, with three levels for scoring: critical literacy level (≥ 28 points), interactive literacy level (21–27 points), and functional literacy level (≤ 20 points). Part 3, which evaluated self-efficacy for the prevention of diabetes, was adapted from previous literature reviews [21, 31]. There were 10 items with three levels of agreement: disagree, uncertain, and agree. The total score was in the range of 0 to 30, and the proportional scores were divided into three levels: high (≥ 24 points), intermediate (19–23 points), and low (≤ 17 points). Part 4 examined self-care behaviors for diabetes prevention and was also adapted from literature reviews [21, 31]. The questions were related to food consumption (20 items), exercise (10 items), stress management and rest (10 items), and diabetes treatment behaviors (10 items), for a total of 50 items. There were three possible answers: rarely practice (< 1 time/week), sometimes practice (1–3 times/week), and regularly practice (> 3 times/week). High-level scores were 80% or more (≥ 120 points), moderate-level scores were between 60 and 79% (90–119 points), and low-level scores were less than 60% (≤ 89 points).

After completing the first draft of the questionnaire, it was checked for accuracy using item-objective congruence and reviewed by three external experts in their respective fields (internal medicine, behavioral health, and public health). The questionnaire was validated using a sample of 30 elderly people with similar background characteristics. To determine the reliability of the questionnaire, the Kuder–Richardson formula was employed (KR20 = 0.82). For Parts 3 and 4 of the questionnaires, Cronbach’s alpha coefficients were 0.81 and 0.80, respectively.

### Data analysis

Statistical analyses were conducted using SPSS (IBM, Armonk, NY, USA). General information was described using mean, standard deviation (SD), minimum (Min), and maximum (Max) values as well as frequencies and percentages. Pearson’s correlation coefficient (r) was used to test correlations between diabetes HL, self-efficacy, self-care behaviors, and medical examination results. A simple linear regression was used to investigate the association between each independent factor (diabetes HL, self-efficacy, and self-care behaviors) and glycemic control among participants. The analysis was then adjusted for sex, age, education, DM duration (years), and smoking and alcohol consumption, all of which have been shown to be significantly associated with FBS or HbA1C.

## Results

The mean age of participants was 67.11 ± 6.60 years (min – max = 60–100). More than half (57.0%) were female (50.5%) and married (57.0%). Regarding education level, 66.4% of the subjects were not educated. In addition, more than half (51.4%) were unemployed, and one-third of the sample (29.0%) had financial difficulties. Most of them had diabetes complications (78.0%), including hypertension (67.6%), hyperlipidemia (45.4%), stroke (10.1%), coronary artery disease (5.8%), chronic obstructive pulmonary disease (4.3%), and chronic kidney disease (3.6%). Only 54.1% of the participants had a caregiver. Their average duration of diagnosis with diabetes was 4.36 ± 2.65 years (min – max = 1–20). Over three-quarters of the subjects (75.6%) attended primary care services from the Subdistrict Health Promotion Hospital (Table 1). Most of the participants were non-smokers (77.3%) and did not drink alcohol (71.5%). Their food preferences, in order, were salty (28.5%), bland (26.3%), sweet/sugary (22.5%), fatty (10.6%), and spicy (6.8%). Also, more than half of the subjects (64.0%) regularly forgot to take their medication or chose to take it only sometimes or one to three times per week.

**Table 1:** General characteristics of participants (n = 414)

Variable			n (%)	Mean ± SD (Min – Max)
Sex				
	Male		205(49.5%)	
	Female		209(50.5%)	
Age (years)				67.11 ± 6.60 (60–100)
	60–69		305(73.7%)	
	70–79		85(20.5%)	
	≥ 80		24(5.8%)	
Marital status				
	Single/Widowed/Divorced/Separate		178(43.0%)	
	Married		236(57.0%)	
Education				
	No		275(66.4%)	
	Yes		139(33.6%)	
Employment status				
	Not employed		213(51.4%)	
	Employed		201(48.6%)	
Perceived financial status				
	Insufficient		120(29.0%)	
	Sufficient		294(71.0%)	
Current smoking				
	No		320(77.3%)	
	Yes		94(22.7%)	
Current alcohol consumption				
	No		296(71.5%)	
	Yes		118(28.5%)	
Favorite food taste				
	Bland		131(31.6%)	
	Salty		118(28.5%)	
	Sweet		93(22.5%)	
	Fatty		44(10.6%)	
	Spicy		28(6.8%)	
DM duration				4.36 ± 2.65 (1–20)
Comorbidity				
	No		91(22.0%)	
	Yes		323(78.0%)	
	Type of disease			
		Hypertension (HT)	280(67.6%)	
		Hyperlipidemia	188(45.4%)	
		Stroke	42(10.1%)	
		Coronary artery disease (CAD)	24(5.8%)	
		Chronic obstructive pulmonary disease (COPD)	18(4.3%)	
		Chronic Kidney Disease (CKD)	15(3.6)	
Medication adherence				
	Never forget to take medicine		75(18.1%)	
	Ever forgot-sometimes (1–3 times/week)		265(64.0%)	
	Ever forget-often (≥ 4 times/week)		74(17.9%)	
Having a caregiver				
	No		190(45.9%)	
	Yes		224(54.1%)	

Table 2 presents the diabetes HL, self-efficacy, self-care behaviors, and medical examination results of the study participants. The mean BMI was 22.67 ± 2.85 kg/m^2^, mean SBP was 141 ± 16 mmHg, mean DBP was 80 ± 8 mmHg, and mean FBS was 108.5 ± 29.5 mg/dL. An HbA1c ≥ 6.5% was found in 50.5% of participants, with a mean HbA1c of 6.58 ± 1.21% and eGFR of 78.3 ± 20.4 ml/min/1.73m^2^. The mean score on the diabetes HL test was 20.94 ± 3.93 points. Participants’ mean diabetes HL scores were 2.94 ± 1.06 points for accessibility skills, 3.50 ± 0.79 points for cognitive skills, 3.79 ± 0.59 points for communication skills, 3.75 ± 0.69 points for decision-making skills, 3.95 ± 0.78 points for self-management skills, and 3.01 ± 0.78 points for media skills. Overall, the level of diabetes HL was functional (52.9%). The mean score for self-efficacy was 20.17 ± 3.08 points, and the majority were of a moderate level (51.2%). In terms of self-care behavior, the mean scores were 98.86 ± 16.91 points, and the scores were a mix of the low (41.1%) and moderate (35.3%) levels.

**Table 2:** Diabetes HL, self-efficacy, self-care behaviors, and medical examination results of participants

Variable			n (%)	Mean ± SD (Min – Max)
Total HL				20.94 ± 3.93 (13–30)
	Functional literacy (scores ≤ 20)		219(52.9%)	
	Interactive literacy (scores 21–27)		149(36.0%)	
	Critical literacy (scores ≥ 28)		46(11.1%)	
	Domain			
		Access skills		2.94 ± 1.06 (2–5)
		Cognitive skills		3.50 ± 0.79 (2–5)
		Communication skills		3.79 ± 0.59 (2–5)
		Decision skills		3.75 ± 0.69 (2–5)
		Self-management skills		3.95 ± 0.78 (2–5)
		Media skill		3.01 ± 0.78 (2–5)
Self-efficacy				20.17 ± 3.08 (16–28)
	Low level (scores ≤ 17)		140(33.8%)	
	Moderate level (scores 18–23)		212(51.2%)	
	High level (scores ≥ 24)		62(15.0%)	
Self-care behaviors				98.86 ± 16.91 (78–198)
	Low level (scores ≤ 89)		170(41.1%)	
	Moderate level (scores 90–119)		146(35.3%)	
	High level (scores ≥ 120)		98(23.6%)	
BMI (kg/m^2^)				22.67 ± 2.85 (16.0–36.2)
	Underweight (< 18.5)		20(4.8%)	
	Normal (18.5–22.9)		235(56.8%)	
	Overweight (23.0-29.9)		92(22.2%)	
	Obesity (≥ 30)		67 (16.2%	
SBP				141 ± 16 (101–186)
	< 140 mmHg		194(46.9%)	
	≥ 140 mmHg		220(53.1%)	
DBP				80 ± 8 (60–106)
	< 90 mmHg		364(87.9%)	
	≥ 90 mmHg		50(12.1%)	
eGFR (ml/min/1.73m^2^)				78.3 ± 20.4 (27.3–120.1)
	G1-Normal (≥ 90)		163(39.4%)	
	G2-Mildly decreased (60–89)		148(35.7%)	
	G3a-Mildly to moderately decreased (45–59)		81(19.6%)	
	G3b-Moderately to severely decreased (30–44)		20(4.8%)	
	G4-Severely decreased (15–29)		2(0.5%)	
	G5-Kidney failure (< 15)		0(0.0%)	
FBS (mg/dL)				108.5 ± 29.5 (56–256)
	Normal (< 100)		222(53.6%)	
	Impaired fasting glucose (100–125)		120(29.0%)	
	Abnormal (≥ 126)		72(17.4%)	
HbA1c (%)				6.58 ± 1.21 (3.50–11.00)
	Normal (< 6.5)		205(49.5%)	
	Abnormal (≥ 6.5)		209(50.5%)	

The test for correlation coefficients between each domain of diabetes HL is presented in Table 3. The highest correlation was found between communication and decision skills (r = 0.782), while the lowest correlation was found between communication and media skills (r = 0.439). Table 4 shows the correlation coefficients between diabetes HL, self-efficacy, self-care behaviors, and medical examination results. There was a high positive correlation between diabetes HL and self-efficacy (r = 0.78), diabetes HL and self-care behaviors (r = 0.76), and self-efficacy and self-care behaviors (r = 0.84). The score of diabetes HL was significantly correlated with BMI (r = -0.197), SBP (r = ‑0.325), and eGFR (r = 0.226). The level of HbA1C was also found to be significantly correlated with BMI (r = 0.246), SBP (r = 0.225), DBP (r = 0.100), eGFR (r = -0.156), and FBS (r = 0.187). The linear regression showed that FBS level was inversely associated with diabetes HL score (Beta = -0.21), self-efficacy (Beta = -0.43), and self-care behaviors (Beta = -0.35) when controlling for sex, age, education, DM duration, smoking, and drinking alcohol (Table 5). HbA1C level was negatively associated with diabetes HL score (Beta = -0.52), self-efficacy (Beta = -0.39), and self-care behaviors (Beta = -0.42).

**Table 3:** Correlation coefficients (r) between each domain of diabetes HL

Health literacy	1	2	3	4	5	6	7
1. Access	1						
2. Cognitive	0.570^**^	1					
3. Communication	0.530^**^	0.659^**^	1				
4. Decision	0.625^**^	0.677^**^	0.782^**^	1			
5. Self-management	0.568^**^	0.674^**^	0.705^**^	0.719^**^	1		
6. Media	0.709^**^	0.674^**^	0.439^**^	0.581^**^	0.598^**^	1	
7. Total HL	0.832^**^	0.845^**^	0.795^**^	0.861^**^	0.843^**^	0.816^**^	1

^**^ Significance at the 0.01 level (2-tailed)

**Table 4:** Correlation coefficients (r) between diabetes HL, self-efficacy, self-care behaviors, and medical examination results

	HL	Se	Be	BMI	SBP	DBP	eGFR	FBS	HbA1C
Total HL	1								
Self-efficacy (Se)	0.781^**^	1							
Behaviors (Be)	0.760^**^	0.836^**^	1						
BMI	− 0.197^**^	− 0.189^**^	− 0.201^**^	1					
SBP	− 0.325^**^	− 0.277^**^	− 0.298^**^	0.212^**^	1				
DBP	− 0.082	− 0.133^**^	− 0.108^*^	0.136^**^	0.332^**^	1			
eGFR	0.226^**^	0.142^**^	0.161^**^	− 0.079	− 0.299^**^	− 0.045	1		
FBS	− 0.178^**^	− 0.381^**^	− 0.338^**^	0.085	0.164^**^	0.060	− 0.085	1	
HbA1C	− 0.392^**^	− 0.369^**^	− 0.400^**^	0.246^**^	0.225^**^	0.100^*^	− 0.156^**^	0.187^**^	1

^*^ Significance at the 0.05 level (2-tailed)

^**^ Significance at the 0.01 level (2-tailed)

**Table 5:** Diabetes HL, self-efficacy, and self-care behaviors associated with glycemic control by linear regression

Outcome	Factor	Model	B	S.E.	Beta	P-value	95% CI	R^2^
**FBS**	Diabetes HL score	Unadjusted	-1.34	0.364	− 0.178	< 0.001	-2.05, -0.62	3.2%
		Adjusted*	-1.54	0.459	− 0.206	0.001	-2.45, -0.64	11.0%
	Self-efficacy score	Unadjusted	-3.64	0.436	− 0.381	< 0.001	-4.50, -2.79	14.5%
		Adjusted*	-4.08	0.483	− 0.427	< 0.001	-5.03, -3.13	22.2%
	Self-care behavior score	Unadjusted	-0.62	0.084	− 0.338	< 0.001	-0.78, -0.45	11.4%
		Adjusted*	-0.64	0.094	− 0.350	< 0.001	-0.82, -0.45	17.8%
**HbA1C**	Diabetes HL score	Unadjusted	-0.12	0.014	− 0.392	< 0.001	-0.15, -0.09	15.3%
		Adjusted*	-0.16	0.018	− 0.524	< 0.001	-0.20, -0.13	23.8%
	Self-efficacy score	Unadjusted	-0.15	0.018	− 0.369	< 0.001	-0.18, -0.11	13.6%
		Adjusted*	-0.15	0.020	− 0.390	< 0.001	-0.19, -0.11	19.1%
	Self-care behavior score	Unadjusted	-0.03	0.003	− 0.400	< 0.001	-0.04, -0.02	16.0%
		Adjusted*	-0.03	0.004	− 0.415	< 0.001	-0.04, -0.02	20.7%

* Adjusted for sex, age, education, DM duration (year), smoking, drinking alcohol

## Discussion

The main goal of this study was to identify associations for diabetes HL, self-efficacy, and self-care behaviors with glycemic control in elderly patients with T2DM. The majority of the participants obtained low scores for diabetes HL. This could be due to the lack of formal education among most participants in the current study. The findings of this study were consistent with several studies which showing that the majority of elderly patients with T2DM had low levels of HL [5, 11, 33]. Moreover, the prevalence of inadequate HL is more common in low-income, highly uneducated elderly patients with diabetes [34]. In addition, this study found that all six dimensions of HL were positively associated with self-care behaviors. Similar to a previous study health literacy dimensions accounted for 28.8% of the total variation in self-care behaviors [35]. A systematic review indicated that the lowest mean health literacy score was for interactive health literacy (compared to functional and critical health literacy) [36]. Interactive health literacy is fundamental for developing the cognitive and social skills needed to participate in social activities and understand information that could improve health behaviors [36]. A similar study concluded that low interactive health literacy among people with diabetes indicated poorer communication skills regarding health information [5]. Moreover, another study showed that low and limited functional health literacy resulted from having less or no education and the deterioration of visual acuity that comes from health complications due to chronic diabetes [16].

When considering glycemic control in elderly adults with T2DM, the current results showed that half of participants (50.5%) had HbA1C levels ≥ 6.5%, with a mean HbA1c of 6.6. Regarding FBS, there was a higher value of 126 mg/dL at 17.4%. The effect of poor regulation of HbA1c levels on the body has been discussed extensively in previous literature reviews. Those studies found that the majority of participants had little or no education, low incomes, and improper medication intake, which may have negative impacts on healthcare behaviors. Similar to many previous studies, this study found that inadequate education and insufficient income were statistically significantly associated with decreased self-care behaviors among people with T2DM, resulting in increased blood sugar levels among them [5, 25, 37]. According to some research, people with low health literacy scored lower on the diabetes awareness test. As a result, they were less likely to control their glucose levels and had lower HbA1c levels than those with higher HL [37].

The self-efficacy of elderly people with T2DM was found to be moderate. Individuals with a higher self-efficacy are more motivated to engage in a behavior because they believe in their own capacity to perform specific tasks; thus, self-efficacy has a positive effect on health behaviors [21]. To promote self-efficacy in diabetes patients, cognitive and social skills must be taken into account, and it should be suitable for the context being studied [25]. This will improve diabetic patients’ cooperation with both prevention and therapy programs [25]. A study on Iranian patients with diabetes found that having low self-efficacy resulted in poor self-care behaviors [16]. Another study found that 16% of the participants had low self-efficacy scores, suggesting that they lacked confidence in diabetes management [25]. In addition, the results of this study revealed an interesting relationship between self-efficacy and health literacy. It showed that health literacy was associated with elderly diabetic people’s self-efficacy to control their blood sugar levels. A previous study showed that health literacy was positively associated with self-efficacy and could be used as an important predictor of self-efficacy in patients with diabetes [25].

Our current study found that elderly participants obtained moderate and low scores for self-care behaviors. According to Bandura, personal factors and experiences can influence an individual’s perceptions, beliefs, and actions [21]. In this study, elderly patients with T2DM tended to have poor dietary habits, especially related to “Eating foods or desserts made with coconut milk, sweet sugary snacks, fried crisps, food using a lot of fish sauce, and oily food such as Hang Lay Curry, Green Curry, Khao Soi, and Sai Oua.” Dietary habits are based on family lifestyle, experience, and culture. In terms of diabetes treatment behaviors, the majority of the participants did not take their medications on time, forgot to take medications, or took medications irregularly, such as only when their blood sugar level was high. The findings were consistent with a study that found that participants had poor self-care behaviors because they were not concerned about the health consequences. In addition, elderly diabetic patients tend to perform self-care behaviors based on their experiences and lifestyle. Therefore, promoting and improving self-care behaviors among these patients should be a priority [5, 25] because self-care behaviors are positively associated with quality of life [30].

Our results also found that self-care behaviors were positively associated with diabetes HL and negatively associated with blood sugar levels. This is consistent with several previous studies indicating that low health literacy is associated with decreased self-care behaviors, resulting in increased glycemic control [5, 38]. Because many elderly patients with diabetes have poor health literacy, they often have difficulty reading drug labels, accessing health information, or understanding their healthcare provider’s suggestions [38]. A previous literature review suggested that health literacy and diabetes knowledge can determine relevant self-management behaviors among patients [37]. Similar to a previous study, health literacy levels were associated with self-care behaviors and diabetes treatment by improving physical behaviors and enhancing health knowledge [39]. Based on these findings, it is critical to develop HL programs in order to improve diabetes prevention care behavior and glycemic control among Thai older adults with DM.

Associations between the health variables BMI, SBP, DBP, eGFR, and HbA1C were observed. According to Pender, individual biological factors and health status are risk factors affecting patients’ health and behaviors, having both direct and indirect influences on their commitment to improve their health behaviors [40]. Moreover, metabolism and physiological functions change with age. Elderly patients, especially those with diabetes and chronic kidney disease, are more likely to live with co-morbidities [41]. A previous study found that when adjusting the variables, BMI and blood pressure were significantly related to glycemic control in diabetic patients [42]. Similarly, a follow-up study of chronic kidney disease patients with T2DM found that SBP and HbA1c were significantly reduced in glomerular filtration pathology [43]. Some studies have found that an inability to control blood pressure, biological risk factors, and creatinine and HbA1c levels are responsible for renal complications among people with T2DM [41, 43].

The final variable which needs to be discussed is diabetes HL. This study found that health literacy was significantly related to FBS and HbA1C. A previous study discovered that inadequate health literacy was an independent predictor of poor glycemic control; moreover, it was associated with a lower likelihood of achieving good control [11]. HbA1C is an objective clinical end point that has been linked to healthcare use and costs [44]. It results in disability and can be life threatening among patients [11]. A study from Brazil found a significant association of health literacy with HbA1c and glycemic control [33]. A study in China also found that higher FBG levels were associated with an increased risk of complications, such as stroke [45]. Health literacy not only limits the ability to read but also the adherence to medical advice. It combines several key skills to enable individuals to gain knowledge, understand that knowledge, and access accurate information in order to promote and maintain good health [13, 33]. These findings suggest that improving diabetes HL and self-care behaviors among older adults with DM in rural communities may help to maintain not only blood sugar levels, but also other health conditions and prevent diabetes complications such as decreased kidney function.

There are some limitations of this study that are worth mentioning. First, this study was cross-sectional, so we are unable to indicate causality or the direction of the associations. Second, this study was conducted in Muang District, Phayao Province, northern Thailand, so our findings may not be representative to other diabetes populations across the country. Additionally, this study highlights the need for caution when interpreting the effects of HL in diverse populations. Thirdly, recall bias may occur as a result of the use of a questionnaire. Some elderly people are unable to provide complete information during data collection because they may not exactly remember their information, such as treatment. To prevent lost or missing data, the researcher reviewed health information from the sub-district hospitals and community health centers in the JHCIS program. Finally, during the process of taking blood samples from the elderly participants, there were some difficulties due to the COVID-19 outbreak, as appointments had to be done early, and the amount of time and number of people had to be limited in order to comply with the government’s pandemic prevention measures. Therefore, the researcher had to remind participants of the procedures prior to conducting the research. Moreover, participants had to adhere to the physical examination guidelines. For future studies, action research should be considered to provide a collaborative approach to analyze and find solutions that are appropriate for the needs of the community and patients with T2DM in the studied context.

## Conclusion

The findings of this study showed that diabetes HL is associated with self-efficacy and self-care behaviors affecting the control of blood sugar in elderly patients with T2DM. The results suggest that medical departments and public health agencies should prioritize health literacy and diabetes-related experience and knowledge among elderly patients in order to motivate and encourage them to pursue the expected outcomes. For instance, accessing health information, understanding the disease and its complications, and communicating with peers and healthcare personnel lead to better decision making and ongoing behavioral changes. Moreover, improving health literacy levels and promoting appropriate self-care behaviors not only enhance health outcomes but also have a positive economic impact, which benefits both individuals and national health systems. Health care providers should focus on building strong relationships between heath network partners and health-related agencies and on the provision of health literacy training programs for glycemic control in elderly patients with T2DM.

## Data Availability

Most of the datasets generated and analyzed during the current study are included in the manuscript, hence, for those who need the underling row data, they can get from corresponding author on reasonable request.

## Abbreviations

HL: Health literacy
T2DM: Type 2 diabetes mellitus
WHO: World Health Organization
HbA1c: Glycosylated hemoglobin
JHCIS: The Java Health Center Information System program
BMI: Body Mass Index, FBG:Fasting blood glucose
eGFR: estimated Glomerular Filtration Rate
HT: Hypertension
CAD: Coronary artery disease
COPD: Chronic obstructive pulmonary disease
CKD: chronic kidney disease.
